# Engineering the RNA-Nanobio Interface

**DOI:** 10.3390/bioengineering4010013

**Published:** 2017-02-15

**Authors:** Vaibhav Murthy, Robert K. Delong

**Affiliations:** Nanotechnology Innovation Center of Kansas State (NICKS), Department of Anatomy and Physiology, Kansas State University, Manhattan, KS 66506, USA; vmurthy@vet.k-state.edu

**Keywords:** RNA-nanoparticle complexes, nano-bio interface, RNA-nanobio interface

## Abstract

RNA nanotechnology is attracting a great deal of attention recently. As the multiple roles that RNA plays in molecular biology and physiological regulation become clearer, there are many opportunities for engineering RNA-Nanoparticle Complexes (RNA-NPCs). The high “engineerability” of RNA-NPCs comes from the ability to modify the RNA and NP chemistry. For example, the NP can be derived from materials with anticancer activity and the RNA delivered by it, designed to target cell signaling pathways that contribute to the molecular basis of these diseases. Despite this rapid advancement and the availability of new quantification and characterization techniques, a key challenge is to develop a better understanding of the RNA-nanobio interface; that is, the interactions of RNA with NP (RNA-nanobio interface) and how that impacts the structure, function, delivery, and activity of the RNA. Here, we attempt to summarize the state-of-the-art in this new and exciting field, and to lay out potential directions for bioengineering research on RNA-NPCs.

## 1. Introduction

**Figure 1:**
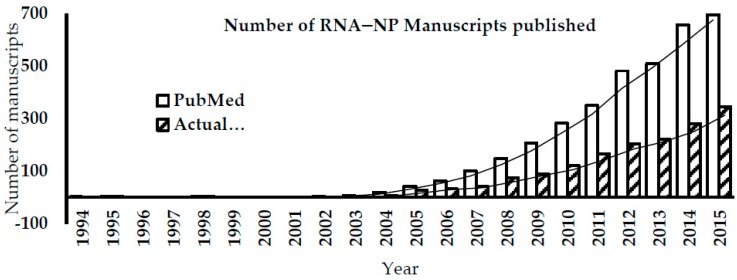
Literature search on the number of RNA-NP manuscripts published (on PubMed) using the keyword “RNA Nanoparticle” (or “RNA-NP”, “RNA-Nanoparticle” variations). The number of actual articles with RNA-NP content is substantially lower than the number from the general published (of RNA-NP) article search.

Nanotechnology has emerged at the forefront of science in the past decade with many applications in engineering. In parallel, with advancements in genomics and proteomics, much of the early biomolecular nanotechnology work was done on DNA or protein-based Nanoparticles (NPs). However, with the advancement of our understanding of RNA, RNA nanotechnology has recently exploded onto the scene (Figure 1). 

As shown in Figure 1, there has been an exponential increase in RNA nanotechnology publications in the last 5–6 years. RNA presents some interesting opportunities in comparison to proteins, where its chemistry is much less complicated, and there is a rich pipeline of modifications to explore in conjunction with NPs. So too, there are new nanomaterials now available from virtually every element within Mendeleev’s periodic chart with a myriad of potential applications. The intent of this review is to summarize the current status of the field, and to identify some of the limitations in nanobio research. 

## 2. Engineering the RNA-Nanobio Interface

RNA is more than just a source code and genetic information [1,2]. While proteins have been classically thought of as the engineers within cells and tissues, or more specifically enzymes, RNA can also catalyze biochemical reactions [2,3]. Using structural motifs, RNA has been developed with specific functions: Riboswitches, Aptamers, Splice-Switching Oligonucleotides (SSO) etc. [2,3]. Also, similar to proteins, RNA has the ability to fold into two- and three-dimensional nano-structures [4,5,6], capable of self-assembly [7,8]. The significant advancements made in understanding RNA chemistry have made RNA therapeutics a highly sought after field. 

Conventionally, RNA-NPs are defined as nanostructures primarily composed of RNA [7,9], with some composed in combination with DNA [5]. The most common example of the latter being pRNA-3WJ, has been extensively researched and characterized [8,9,10,11,12,13,14]. Here, we primarily consider RNA-molecules interacting with core NP (RNA-NPC, RNA-Nanoparticle Complexes) made of organic or inorganic NPs. 

RNA-NPCs have been used successfully as bio sensors [15,16,17]—to detect cancerous cells, as antibacterial-[18,19], and anti-cancer [20] agents. NPs have also been used extensively as vector-systems for delivery of RNA to cells and tissues [20,21,22,23,24,25,26,27,28,29,30,31], even as early as 1998 [32]. Addition of RNA substantially alters the surface properties and physical behavior of NP. A variety of approaches have been used to modify the NP surface before or after RNA interaction [33,34]. 

**Figure 2:**
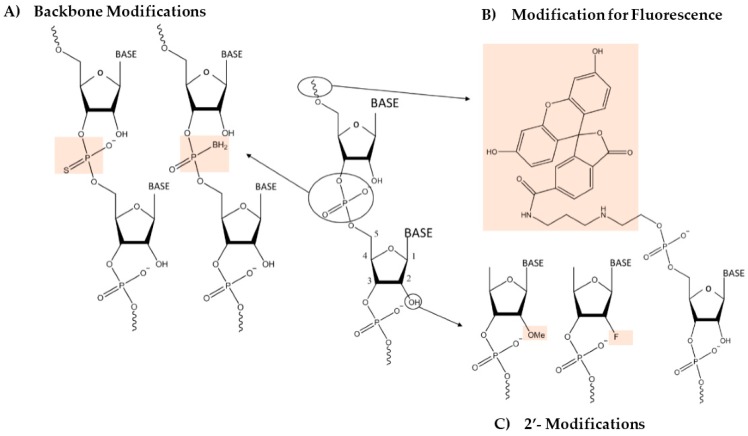
Some examples of RNA chemical modifications which can now be synthesized due to the availability of various synthons compatible with automated nucleic acid synthesizers. (**A**) Modifications in the phosphodiester backbone such as phosphorothioate (S=P)-or-boranophosphate (BH_2_-P); (**B**) Modifications (most commonly at 5′ and 3′) with fluorescence dyes (5′-Fluorescein or 6-FAM in figure) allow for visual detection of RNA-NPCs (RNA-Nanoparticle Complexes), since RNA does not possess intrinsic fluorescence; (**C**) Modifications in the ribose such as in the 2′ position (O-Me, -F and others or ring modified versions).

The engineerability of the RNA-nanobio interface arises in large part from the ability to specify the interactions between the RNA molecule and the NP surface. This is achieved through chemical manipulations of the RNA molecule or through chemical modifications on the surface of the NP. In the case of RNA, a variety of chemistries are available which modify either the backbone or the base. Additionally, the 5′ or 3′ termini of the RNA molecules can be synthesized with various derivatives which impart desirable properties on them such as fluorescence or stability (Figure 2). 

As shown in Figure 2, a wide variety of modifications are available today for RNA in the backbone or linkage, nucleotide base and sugar. Only a few are shown but it is important to point out that these were primarily developed for increased stability and resistance to nuclease digestion—important for therapeutic applications. Thus, the impact of these chemistries on NP interaction and nanobio activity is almost totally unknown. 

*RNA Phosphodiester backbone modifications*: The phosphodiester bond between RNA is a hotspot for stability manipulations. Some of the more common of these, that are likely to impact the RNA-nanobio interface are the Phosphorothioate (PS) and boranophosphate [35] derivatives, which are seen to have no additional toxicity effects [36]. Both of these provide stability to the RNA and resistance against nuclease degradation. 

*RNA ribose sugar modifications:* A second hotspot for modifications is on the 2′ carbon of the ribose sugar molecule. 2′-O-Methyl and 2′-F modifications are seen to substantially improve RNAse resistance. However, 2′-O-Me synthesized modified oligos are not recognized by other enzymes. 2′-F oligos, are now being extensively used due to their ability to retain native RNA configurations [37].

*RNA modifications for fluorescence*: RNA possesses no intrinsic fluorescence. Some RNAs such as aptamers may need to target the cell membrane, others such as antisense and siRNA act in the cytosol, whereas SSOs and other RNAs designed for transcription modulation require nuclear delivery. Here, it can be helpful to visualize cytosolic localization by modifying the RNA with fluorescent moieties. For example, carboxyfluorescein (6-FAM) and Cy5 modifications to the RNA can be used to view the RNA location in cell imaging. Additionally, conjunction of the dyes on either end of RNA has been used as a primitive cleavage marking system [26] in the case of ribozymes. Dye labeling has also been extensively used in electrophoretic mobility shifts to see specific protein interactions [38]. More recently, fluorescent RNA aptamers have been generated using SELEX (Systematic Evolution of Ligands by Exponential Enrichment) to mimic common fluorescent tags. Aptamer Spinach (mimics GFP) has been successfully used in visualizing live cells [39]. Similarly, aptamer Mango (orange fluorophore) has been developed with increased fluorescence [40]. Self-assembling RNA-NP nanocubes functionalized with malachite green aptamer have been used to monitor the correct self-assembly of 3-D nanostructures. Fluorescence is only observed with successful formation of the nanocube [5]. Fluorescent aptamers are advantageous compared to conventional dyes due to their lower toxicity and enhanced permeability. The use of fluorescent aptamers with inorganic nanoparticles, however, has not been studied extensively, and remains an area of potential development. 

**Figure 3:**
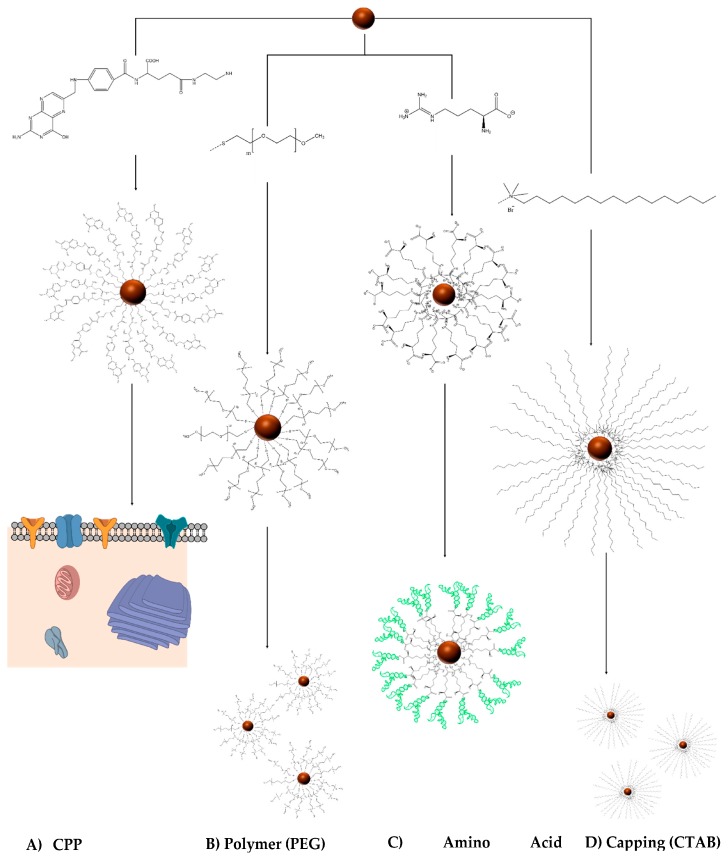
Examples of NP (Nanoparticle) modifications. (**A**) Cell-penetrating peptide-decorated NPs enhance cellular uptake of the NP (**B**) Polymer modifications such as PEG (polyethyleneglycol) (in figure) and PEI (polyethyleneimine) enhance NP stability by preventing aggregation in solution; (**C**) Amino Acid modifications such as arginine or lysine improve RNA binding to NP. Certain aptamers are synthesized with specific amino acid recognition motifs; (**D**) Surfactant capping modifications such as citrate, CTAB (cetyltrimethylammonium bromide) etc., have been used to restrict NP size, prevent aggregation, and improve stability in solution.

In addition to engineering the RNA, many investigators modify NP chemistry. NP (organic, inorganic, nucleic acid or liposome) surface modifications have been used to confer beneficial properties to NPs (Figure 3). Below are some of the most common modifications used across the board for liposomes, polymer NPs or inorganic NPs. 

*NP modifications with polymers*: One of the prevailing types of common functionalization is polyethyleneglycol (PEG) modification, generally thought to confer physical stability upon the NP and prevent aggregation [41]. Polyethyleneimine (PEI) has been seen to improve binding of siRNA with electrostatic interactions on gold NPs, enabling retention of native RNA properties [42]. Chitosan, another polymer, has also been used to functionalize NPs to improve stability. Chitosan coated NPs possess high positive Z-potential, and are repelled by electrostatic forces, preventing aggregation in solution [43]. 

*NP modification with amino acid*: NPs can be functionalized with amino acids, such as arginine, to enhance binding of RNA. It is well known that RNA motifs can be engineered to have specificity for certain amino groups [44,45,46]. Similarly, based on binding strength, aptamers have been created with amino acid specific recognition motifs [46,47]. Amino acid decorated NPs have been used to bind to RNA with improved efficacy, enhancing distribution and stability. 

*NP modification with cell-penetrating peptides (CPP)*: NP uptake into cell is crucial for cancer targeting. Cell-penetrating peptide functionalized NPs have been used with some success to enhance NP uptake. CPP modified liposomes, however, were seen to have inefficient RNA unloading post internalization; polymer NPs, however, did not have the same effect [48,49]. 

*NP surfactant capping*: NP growth can be controlled with capping agents such as Citrate, Cetyltrimethylammonium Bromide (CTAB), Oleic Acid (OA) etc. Capping agents enable uniform homogenous dispersion of NPs in colloidal solutions. Additionally, capping agents act as stabilizing agents by preventing NP aggregation. However, their distribution on the NP surface also inhibits catalytic active sites, preventing accurate reactions [50].

*NP modifications with chemical functional groups*: Alternatively, water-soluble NPs are modified with chemical functional groups such as carboxylic acids (that prevent aggregation due to electrostatic repulsion) or thiols [34]. Thiols in particular, are seen to preserve inorganic NP core stability against heat and aggregation [51]. This has been utilized extensively for gold NPs, since thiols have high affinity for gold. 

*Miscellaneous NP modifications and targeting*: Sugar molecules often decorate the surface of cells and tissues and are conjugated to proteins to form glycoproteins. Glycosaminoglycans of various forms and sizes are present within the extracellular matrix and form the bulk of non-proteinaceous material present therein and are widely used as biomedical materials such as heparin and many others. This represents an important targeting opportunity where, for example, one study used 2-deoxy-D-glucose modified polymer NPs to target intracranial tumors which were shown to better penetrate the blood–brain barrier and accumulate in intracranial tumors [52]. Here, aptamers can be screened and optimized for binding to these targets in order to direct the NP to these sites of disease.

## 3. Characterizing the RNA-Nanobio Interface

Another opportunity represents the techniques necessary to characterize the RNA-nanobio interface. Engineering being principally mathematically driven, it is important to be able to have quantitative parameters by which to optimize the performance of RNA-NPC. Today, a suite of technologies has become available which can shed light on the interaction of the RNA with the surface of the NP. For example, innovations in a variety of different microscopy approaches now allow us to essentially glimpse the surface. Common techniques include microscopy: Atomic Force Microscopy (AFM), Scanning Electron Microscopy (SEM), Transmission Electron Microscopy (TEM) and others. Surface Plasmon Resonance (SPR) properties of NPs (only Noble metals-Au and Ag), based on free electron oscillations-which are size and shape dependent [53], may be useful in characterizing the RNA nanobio interface. 

Zeta potential (ζ, ±) and Dynamic Light Scattering (DLS) are two common techniques which reveal changes in the electrostatic potential at the surface when RNA binds or in the hydrodynamic diameter of the particle respectively. These techniques, while semi-quantitative, tend to be difference measurements (positive to negative, or shift in peak) and may not generate information that ultimately correlates with bio-activity. 

Techniques which can discriminate the nature of the nanobio interaction include nuclear magnetic resonance (NMR), infra-red (IR), ultraviolet (UV) spectroscopy and others. Further, surface functionalization characterization can be studied by ^1^H NMR, Raman Spectroscopy and Fourier Transform Infrared Spectroscopy (FT-IR). Based on the functional groups present, the peaks obtained from the NMR and IR can be used to detect the presence and binding of groups on the NP surface. Additionally, shifts in NMR and IR peaks can be used to detect conformational changes caused due to NP binding. Techniques such as X-ray Photon Spectroscopy (XPS) have been used with some success in determining the configuration of RNA on NP surfaces [54,55]. For some biomedical nanomaterials, certain NPs are fluorescent. The unique spectral signature of NPs shifts when bound to RNA can be used to determine qualitatively if the RNA-NP complex has been formed. This technique, developed by our group, Two-Dimensional Fluorescence Difference Spectroscopy (2D-FDS) [56], provides another potential quantitative parameter with which to correlate RNA-NPC engineering and design principles with bio-activity.

Thermal characteristics of self-assembling RNA-NPs have also been used to characterize the RNA-nanobio interface. The melting point temperature of self-assembling RNA-NPs has been used to determine controlled release as well stability over temperature ranges [5,9,57]. Further, addition of RNA causes changes in the melting temperature of the RNA-NP complex [57]. 

Table 1 is a summary of examples of RNA nanobio characterization where a variety of RNA types such as siRNA, aptamer, microRNA (miRNA) and ribozymes have been studied.

## 4. RNA-NPC and the Biological Milieu

NPs possess physiological and chemical properties that are different than their parental bulk materials. Addition of RNA and functionalization further change the NP surface properties and how they interact with the biological milieu. 

**Figure 4:**
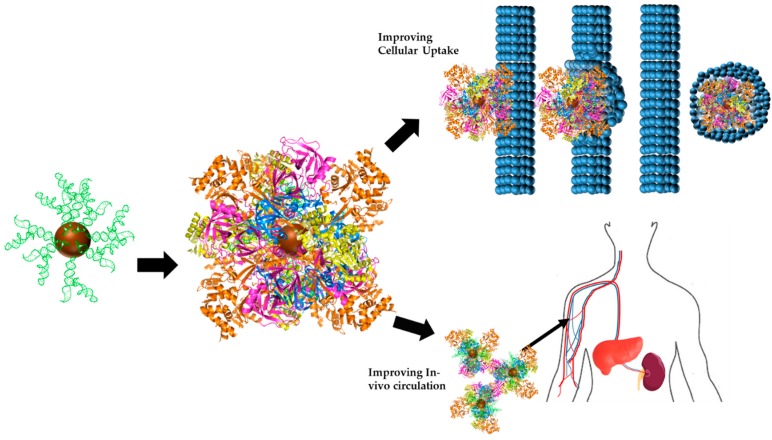
Protein Corona agglomeration causes changes in the identity of the RNA-NPC therapeutic. Interactions with proteins can cause masking of the RNA of interest, rendering the RNA-NPC ineffective.

RNA-NPCs introduced into biological fluid have been seen to form Protein Coronas (PC)—aggregations of proteins on the NP surface [63]. These PCs alter the properties of NPs and affect cellular uptake, half-life and distribution amongst other properties [64]. The formation of the PC on the NP surface (in protein-NPs) has been harnessed to be advantageous, as shown in Figure 4. 

Intravenous injections of NP drugs necessarily requires an understanding of the interactions between RNA-NPC and blood components-PC accumulation, some of which possess RNAse activity and have been seen to hydrolyze RNA [65]. In cases of in vivo studies, understanding the adsorption of blood proteins to the RNA-NPC surface is crucial. For instance, decrease in proteins adsorbed to the NP surface allows for increased RNA loading for a variety of purposes. Moreover, decreasing the protein adsorption can improve circulation half-life and unwanted host responses [66]. Understanding the interactions of PC proteins with the RNA-NPC surface, additionally, is crucial to determine drug toxicity, dosage, and interactions with off-target materials. 

NP-mediated therapy holds the potential for rapid advancement in diagnosis in treatment; however several shortcomings need to be overcome. Firstly, RNA is highly labile and chemically unstable, rendering it extremely sensitive to RNAse degradation. This becomes of special importance for intra-venous drug applications, as the RNA is susceptible to hydrolysis. In addition, the lack of specificity renders it prone to off-target effects due to partial matching. There have been efforts to curb these with structural modifications [33] or using NP as vector systems [33,35]. 

RNA, unlike proteins, does not possess intrinsic fluorescence. In such cases, the intrinsic fluorescence properties of certain NPs (e.g., Quantum dots and under certain conditions, Gold and Silver) have been used to visualize RNA-NPCs. Additionally, the RNA or NPs have been functionalized with dyes for fluorescence [14,20,57,58,59]. Even with these advancements, however, fluorescence for visualization still remains a shortcoming for numerous RNA-NPCs without intrinsic fluorescence. Finally, characterization of the RNA-nanobio interface and its interactions with the PC remain elusive. Even with the development of techniques such as 2D FDS, understanding the molecular interactions at the RNA-nanobio interface still remain a mystery. 

## 5. Conclusions

In brief, developing a better understanding of the RNA-nanobio interface is critical to engineering better RNA-NPCs. With the myriad of chemistries available both for RNA and NP, it is now crucial to identify optimal RNA-NPC combinations that will ensure enhanced stability, biocompatibility, and therapeutic effect. Advances in RNA-nanobio characterization are tied to successful RNA-NPC engineering. These opportunities and the challenge of a complex and variable biological milieu in which the RNA-NPC is expected to perform will be important to address for the long-term of RNA-NPC therapeutics. 

## Figures and Tables

**Table 1:** Characterizing the RNA-Nanobio interface.

RNA+ Modification		NP+ Modification	RNA-Nanobio Characterization	Biological Significance	Ref.
Anti-Sense Oligonucleotides (ASO)	-	*Liposome* DOTAP:DOPE-DOC-PEI ^1^	^1^H NMR, FT-IR, Z-potential (ζ)	Selectively target CD33 positive Kasumi-1 cells Greater transfection efficiency Highest downregulation of R2 Treated mice-increased tumor inhibition	[22]
	2′-O-Met, FAM	*Polymer* PLGA + Chitosan	AFM (Atomic Force Microscopy)	Increased cellular uptake by primary lung cancer cells and fibroblasts. Inhibition of telomerase activity Decreased cytotoxicity	[21]
	PNA	*Inorganic* Mesoporous Silica (MSNP) + FITC + SS	SEM, TEM, FT-IR, Z-potential, MALDI-TOF MS ^2^, UV Abs.	Higher cellular uptake Directed delivery into cytoplasm of HeLa cells Silencing of Bcl-2 protein expression Low toxicity	[23]
	TcDNA + PS And B-PPMO	*Nucleic Acid* Self-Forming micelle nanoparticle	TEM, DLS (Dynamic Light Scatter), Critical Micelle Concentration (CMC), Z-potential	ASOs had increased inclination to self-assemble into nanoparticles Fucoidin Sulfate and Dextran sulfate competitively inhibited B-PPMO uptake by Class A scavenger receptor subtypes (SCARAs)	[58]
Aptamer	Apt1 (anti-CD44)+ 2′-F	*Liposome* DPPC:Cholesterol:DSPE+ Maleimide + PEG	Z-potential, Gel electrophoresis	Enhanced CD44 binding Increased cellular uptake by Lung (A549) and Breast Cancer (MDA-MB-231) Cell lines ^3^. Constant IL-6, IL-1β, IL-8—does not induce inflammatory response	[20]
	anti-PSMA ^4^	*Polymer* PLA-PEG-COOH	Z-potential, SEM	PMSA specific Increase in efficiency and selectivity	[24]
	anti-PSMA A9	*Inorganic* Gold+ Thiol+ hexa(ethylene glycol)	DLS, Fluorescence	Selectively labels PSMA positive cells (LNCaP) Stability preserved	[25]
	Endo28 ^5^ Aptamer	*Nucleic Acid* pRNA-3WJ	Serum Stability with polyacrylamide gel, Z-potential, DLS	RNA-NP showed increased binding to AnnexinA2 expressing cancer cells-IGROV-1 RNA-NP w/dox specifically delivers to AnnexinA2positive cell lines. Enhanced AnnexinA2 tumor selective binding	[59]
microRNA	miR-122 mimic	*Liposome* DODMA-eggPC-chol-PEG	Z-potential	Preferentially taken up by tumor cell lines Down-regulation miR-122 genes in tumor tissue. Z-potential in different pH-colloidal stability	[30]
	Anti-miR-155 PMO and PNA	*Polymer* PLGA+ argCPP	BCA Assay ^6^, Flow Cytometry, TEM	Inhibition of miRNA Altered splicing to produce Mcl-1S isoform opposed to Mcl-1L isoform	[60]
	miR-145	*Inorganic* Gold+ thiol oligo	Z-potential, UV spectrophotometry	Overexpression of ectopic miR-145 in PC3 and MCF7 cell types. Efficient delivery system	[61]
	Anti-miR-21	*Nucleic Acid* pRNA-3WJ with PMSA aptamer+ Cy5/Alexa_647_	Z-potential, hydrodynamic diameter, Temperature Gradient Gel Electrophoresis (TGGE)	Specific delivery of anti-miR-21 to LNCaP-FGC (PMSA+) cells Delivery of anti-miR-21 is achieved through PMSA aptamer binding Increase in Caspase III indicating cell death Specific targeting and accumulation of RNA-NP to xenograft tumor Low toxicity profiles in kidney and liver Increase in PTEN and PDCD4 tumor suppressor genes	[62]
Ribozymes	Rzs	*Polymer* PEG-b-PLL	DLS, TEM, EtBr Displacement Assay ^5^, ^1^H NMR, Electrophoretic Mobility Shift	Stable complexes formed Stable in RNAse-rich environment	[27]
	MGMT ^7^+ Fluorescein+ 5′Cy5	*Inorganic* Gold SNA	Gel elec, RT-PCR, DLS, Z-potential	Cleaved MGMT substrate Sustained stability in harsh enzymatic environment Knocked down MGMT in T98G glioma cells Increase in Caspase-3/7 activity	[26]
siRNA	Notch1-homo-siRNA-FAM	*Liposome* DMAPA	Z-potential, TEM, Gel electrophoresis	Protect against RNase A in serum No SKOV3 cell cytotoxicity Increased cellular uptake Increased percentage of apoptotic cells	[29]
	anti-survivin siRNA	*Polymer* PA-PEI + Fe_3_O_4_ Magnetic NP	FT-IR, Z-potential, Gel retardation assay	Increased cell uptake Increased knockdown of survivin gene Increased apoptosis: 3-fold	[31]
	VEGF siRNA/ B-cell lymphoma siRNA	*Inorganic* Gold+ RGD-PEG-COOH Dendrimer	Gel retardation assay, DLS, Z-potential	Delivered specifically to integrin-overexpressing cells Induce specific silencing of genes High transfection efficiency Down-regulation of VEGF and Bcl-2	[28]
	FASE siRNA+ Bcl-xl/2 strand ^8^+ Cy3	*Nucleic Acid* DNA Nanocube-prism+ Cy5	UV-visible melting, UV-visible spectrophotometry for stability, DLS,	Controlled release of siRNA Enhanced stability in DMEM+ serum Increased half-life of 12 hours	[57]

^1^ DOC-PEI conjugate was formulated and incorporated into the liposome to facilitate endosomal release of ASO; ^2^ Matrix-Assisted Laser Desorption/Ionization Time-Of-Flight Mass Spectrometry; ^3^ Both these have been shown to have high CD44 expression ; ^4^ Prostate Specific Membrane Antigen ^5^ Measures surface density of NP with modifications; ^6^ Used to determine the stability of NPs formed. Depending on the weight ratio of NP: Rzs- low weight ratio, there is only slight exclusion of EtBr showing low complexation of NP-RNA; ^7^ O^6^-methylguanine-DNA methyltransferase (MGMT); ^8^ Fatty Acid Synthase siRNA was flanked by DNA spacers and Bcl-xl/2 RNA sequences were added as recognition sequences.
